# Next generation bone tissue engineering: non-viral miR-133a inhibition using collagen-nanohydroxyapatite scaffolds rapidly enhances osteogenesis

**DOI:** 10.1038/srep27941

**Published:** 2016-06-14

**Authors:** Irene Mencía Castaño, Caroline M. Curtin, Garry P. Duffy, Fergal J. O’Brien

**Affiliations:** 1 Tissue Engineering Research Group, Department of Anatomy, Royal College of Surgeons in Ireland (RCSI), 123 St. Stephens Green, Dublin 2, Ireland; 2 Trinity Centre for Bioengineering, Trinity College Dublin (TCD), College Green, Dublin 2, Ireland; 3 Advanced Materials and Bioengineering Research (AMBER) Centre, RCSI & TCD, Dublin 2, Ireland.

## Abstract

Bone grafts are the second most transplanted materials worldwide at a global cost to healthcare systems valued over $30 billion every year. The influence of microRNAs in the regenerative capacity of stem cells offers vast therapeutic potential towards bone grafting; however their efficient delivery to the target site remains a major challenge. This study describes how the functionalisation of porous collagen-nanohydroxyapatite (nHA) scaffolds with miR-133a inhibiting complexes, delivered using non-viral nHA particles, enhanced human mesenchymal stem cell-mediated osteogenesis through the novel focus on a key activator of osteogenesis, Runx2. This study showed enhanced Runx2 and osteocalcin expression, as well as increased alkaline phosphatase activity and calcium deposition, thus demonstrating a further enhanced therapeutic potential of a biomaterial previously optimised for bone repair applications. The promising features of this platform offer potential for a myriad of applications beyond bone repair and tissue engineering, thus presenting a new paradigm for microRNA-based therapeutics.

Bone grafts are second only to blood transfusions on the list of transplanted materials worldwide at a global cost to healthcare systems valued at over $30 billion every year^1^. Standard repair strategies include autografts and allografts but are associated with a number of concerns such as limited tissue volume, risk of rejection as well as chronic pain^2^. Alternatively, the field of tissue engineering aims to regenerate damaged tissues, instead of replacing them, by developing biological substitutes that restore, maintain or improve tissue function. The field relies extensively on the use of stem cells in combination with porous 3D scaffolds that house the cells and provide the appropriate environment for the regeneration of tissues and organs. To enhance their regenerative potential, scaffolds can be specifically tailored to serve as localised delivery depots that release therapeutics in a controlled manner to further enhance bone healing.

The emerging field of RNA interference (RNAi), in the form of microRNAs (miRNAs), offers potential in the novel development of next generation bone tissue engineering therapeutics based on their ability to influence stem cell fate decisions^3^. miRNAs are approximately 22 nucleotides long in their mature form and have the ability to silence protein expression. These miRNAs offer distinct therapeutic advantages in comparison to other nucleic acid therapeutics as the imperfect target binding of miRNAs allows for a multi-targeting effect on complex signalling pathways^4^. Application of miRNAs thus increases the number of avenues that can be manipulated simultaneously, potentially incurring an enhanced therapeutic outcome. A range of synthetic options have been developed to both imitate and inhibit miRNA function, i.e. mimics and antagomiRs, which widens the therapeutic interest in miRNAs to ultimately knockdown or enhance levels of the protein target respectively^4^. Consequently, a number of areas of regenerative medicine, including bone regeneration–the particular focus this study, have recently explored the role of a series of miRNAs by inhibiting or mimicking their function, thereby generating interest in their therapeutic use for tissue repair^5^,^6^.

A critical consideration when applying RNAi to tissue repair relates to the need for the combined application of a delivery vector and a 3D scaffold in order to locally trigger a temporal therapeutic effect. Delivery vectors, typically nanoparticles, mediate uptake of the RNAi cargo, but rapid *in vivo* clearance of nanoparticles from target locations, owing to their small size, limits their local effects to occur over short time periods^7^. Hence, the incorporation of nanoparticles in clinically-translatable 3D scaffolds -designed for tissue regeneration- as miRNA delivery systems holds great promise to fully realise the therapeutic potential of miRNAs for tissue engineering applications^8^.

Recently, 3D scaffolds incorporating viruses belonging to the baculovirus and lentivirus families have showed promising functional delivery of pre-miRs and miR-inhibitors to adipose derived stem cells (ASC) of human and rat origin respectively, in both cases achieving a noteworthy effect in the repair of bone defects *in vivo*^9^,^10^. However, clinical applicability of viral-based miRNA delivery methods is still limited by the threat of adverse immune responses in patients and the risk of insertional mutagenesis^11^. Alternatively, non-viral commercial lipid-based delivery vectors have recently been incorporated in 3D platforms to enhance miRNA-mediated osteogenesis in rat^12^, mouse^13^ and human bone marrow mesenchymal stem cells (hMSCs) respectively^14^. However, cell membrane damage associated with the detergent effect of lipid vectors has been referred to as a limiting factor for their clinical application^15^. With this in mind, we turned to ceramic-based hydroxyapatite nanoparticles (nHA) as vectors, focussing on their major advantage, that is proven high biocompatibility^16^. nHA offers additional advantageous properties for use in bone regeneration applications due to the chemical mimicry to the inorganic component of bones, as well as its demonstrated osteo-conductive and osteo-inductive properties *in vitro* and *in vivo*^17^,^18^. Recent work from our laboratory has developed an innovative bioactive 3D scaffold system for miRNA delivery and extensively characterised its structural, biological and functional properties, including dose-response uptake studies by flow cytometry^19^. This system, with excellent efficiency for the delivery of both reporter miR-mimics and inhibitors (antagomiRs) to hMSCs^19^, incorporates non-aggregating nHA particles as non-viral vectors embedded within a collagen (coll)-nHA porous scaffold developed in our lab specifically for bone repair^17^,^18^,^20^. While composite porous scaffolds containing ceramic and collagen components have shown potential in bone tissue engineering^20^, the need to incorporate additional therapeutic cues within these materials is a recurrent hot topic in the field^21^. Thus, the overall aim of this study was to apply these composite porous scaffolds to deliver miRNA-based therapeutics, in order to enhance osteogenesis by hMSCs for the first time.

A small number of studies have focussed on unravelling the role of miRNAs in regulating osteodifferentiation^5^,^6^. Recent research efforts have identified a selection of miRNAs as osteo-therapeutics, many of which have direct targets that only play a secondary role in the osteogenesis pathway^22^. In contrast, miR-133a has been identified as a direct negative regulator of the master transcription factor of osteogenesis, Runx2^23^; hence, the direct relationship between miR-133a levels and Runx2 expression provides a possibility to target a central activator of osteogenesis. Thus in this study, the central regulatory role of miR-133a was speculated to offer a significant therapeutic target. It was hypothesised that localised inhibition of miR-133a levels in hMSCs would increase Runx2 when using the custom coll-nHA delivery system. Hence, it was proposed that this novel approach, focussed on targeting the key transcription factor of the osteogenesis pathway using miRNA technology in hMSCs for the first time, may allow a promising enhancement of their osteogenic potential (Fig. 1).

In this study, we specifically aimed to develop the first non-viral, non-lipid 3D miRNA-delivery platform using nHA particles to deliver the therapeutic antagomiR-133a within coll-nHA scaffolds engineered specifically for bone repair^24^. This system was capable of manipulating Runx2 levels in hMSCs, importantly using low miRNA doses (20 nM) compared to the literature^19^. This study thus showcases the ability of antagomiR-133a, complexed with nHA particles in a formulation termed nanoantagomiR-133a, to significantly enhance Runx2 levels and osteogenesis in hMSC monolayer as well as to produce a rapid pro-osteogenic effect in hMSCs in 3D culture platforms.

## Results and Discussion

### Intracellular miRNA level analysis in hMSC monolayer osteogenic culture

**Figure 2:**
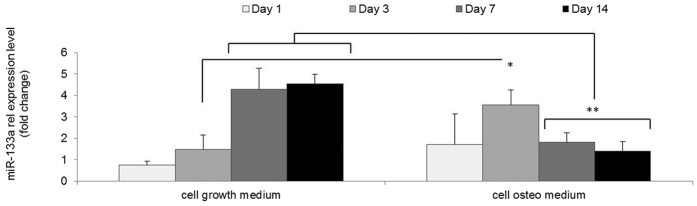
qRT-PCR analysis of miR-133a role in hMSC osteogenesis. Comparison of miR-133a intracellular levels between cells cultured in standard growth medium versus osteogenic media over the course of 14 days demonstrated a natural decrease in miR-133a at later timepoints in osteogenic culture. Mean + standard deviation, n = 4, *p < 0.05, **p < 0.001.

**Figure 3:**
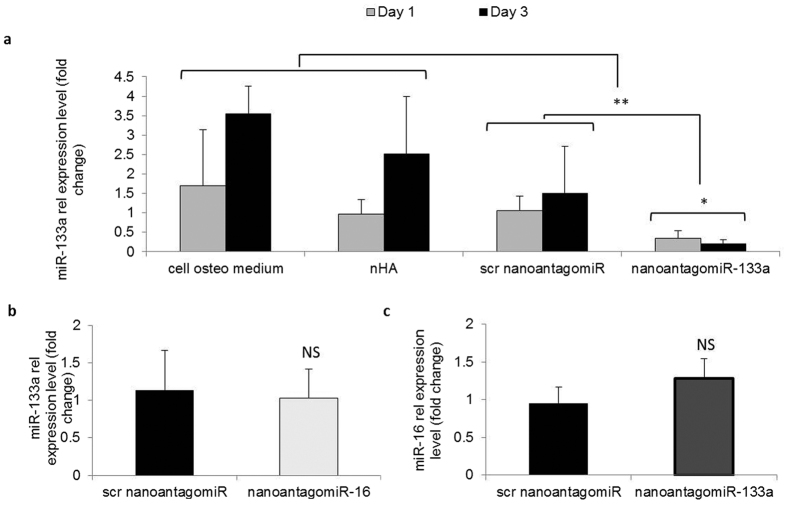
qRT-PCR analysis of miRNA manipulation in hMSC monolayer cultured in osteogenic medium. (**a**) NanoantagomiR-133a treatment demonstrated a maintained functionality with high silencing of miR-133a intracellular levels in hMSC monolayer osteogenic culture. (**b**) NanoantagomiR-16 treatment did not modify intracellular miR-133a levels and (**c**) nanoantagomiR-133a treatment did not modify intracellular miR-16 levels demonstrating treatment specificity and indicating no RISC overloading associated effects. Mean + standard deviation, n = 4, *p < 0.05, **p < 0.001, NS = not significant variation.

The pattern of miR-133a expression during osteogenesis has not been previously assessed in hMSCs. Hence, we sought to elucidate miR-133a expression levels in hMSCs over the course of 14 days comparing standard and osteogenic culture (Fig. 2). miR-133a expression continuously increased in the standard (no osteogenic supplements) culture group, whereas the osteogenic culture group showed a peak at day 3 but reduced levels at the later timepoints of days 7 and 14. This pointed to a link between suppression of miR-133a and progression of *in vitro* osteogenesis, in accordance with previous reports for C2C12 mouse myoblasts and primary mouse vascular smooth muscle cells^23^,^25^. Interestingly, with the nanoantagomiR-133a treatment, a sustained downregulation of miR-133a was obtained from day 1, the earliest time point assessed, which demonstrated accelerated downregulation of miR-133a levels in comparison to untreated cells in osteogenic culture (Fig. 3a). This confirmed the ability of the nanomiR system to achieve high, maintained silencing effects with a functionality level over 80%, which is consistent with that previously reported for this non-viral system^19^. In summary, this data highlighted the role of miR-133a in hMSC osteogenic differentiation and the potent ability of the nHA particles to act as non-viral delivery vectors for specific manipulation of intracellular miRNA levels.

Key issues for miRNA-based therapeutics are silencing specificity and deregulation of the RNA-induced silencing complex (RISC) machinery by overloading. In order to assess specificity and RISC overloading associated effects, the level of miR-16, which is basally expressed in stem cells, was determined following nanoantagomiR-133a treatment (Fig. 3b). Complementary to this, treatment with nanoantagomiR-16 as a negative control was introduced to control for specific manipulation of miR-133a levels. Results in both cases showed no changes in comparison with the reference group, which received the scrambled (scr) nanoantagomiR treatment (Fig. 3b,c). Scr miRNA sequences lack canonical targets in the studied species and thus are commonly utilised as a negative control for miRNA manipulation experiments. This data was thus indicative of specificity and no RISC overloading effects on hMSCs following nanomiR treatment.

### Levels of target and osteogenic markers along with mineral deposition was increased in nanomiR-treated hMSC osteogenic monolayer culture

**Figure 4:**
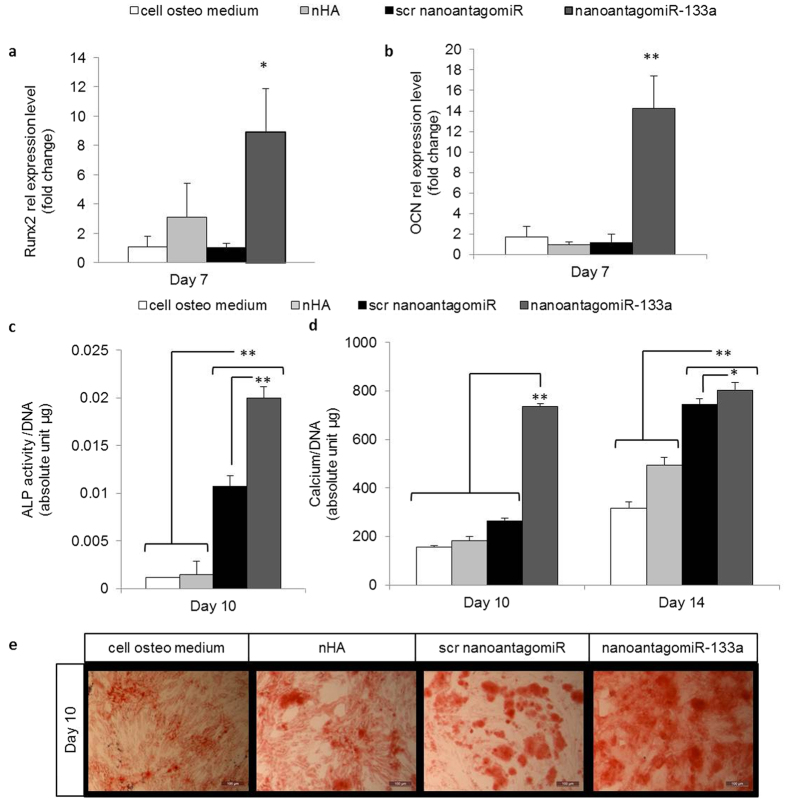
NanoantagomiR-133a treatment enhanced osteogenesis markers in hMSC monolayer. (**a**)Runx2 mRNA expression and (**b**) OCN mRNA relative level were increased in the nanoantagomiR-133a group after 7 days. (**c**) Significantly increased ALP activity levels were found in the nanoantagomiR-133a group 10 days after treatment. Mean + standard deviation, n = 3, **p < 0.001. (**d**) Calcium deposition was markedly increased by day 10 and maintained increased calcium levels compared to the control groups at 14 days after treatment with nanoantagomiR-133a in osteogenic culture. (**e**) Alizarin red staining showed calcium deposits at 10 days after treatment, scale bar = 100 μm. Mean + standard deviation, (**a,b**) n = 4, (**c,d**) n = 3, *p < 0.05, **p < 0.001.

To evaluate the effect of downstream direct targets as well as the osteogenesis achieved with the nanomiR treatment in monolayer, relative mRNA levels were analysed at day 7. Runx2 expression was increased 9-fold with a single dose of the nanoantagomiR-133a treatment (Fig. 4a) while a 14-fold increase was found when osteocalcin (OCN) levels were analysed (Fig. 4b). Of relevance, miRNA induced effects in protein levels are attributable to changes in mRNA expression in up to 84% of the genome^26^,^27^. Additionally, several miRNAs have reported fine-tuning changes (≤3 fold) in the mRNA levels of Runx2 and OCN^28^,^29^,^30^,^31^; in contrast, the higher amplitude changes observed in our work may be associated with a phenotype-switching role^32^, more beneficial to trigger enhanced functional osteogenesis. To further assess functional osteogenesis, alkaline phosphatase (ALP) activity and calcium deposition in the extracellular matrix (ECM) were determined and normalised to double stranded (ds)DNA levels, as a surrogate for cell number. Significantly enhanced ALP activity was found in the nanoantagomiR-133a treatment, corresponding to a 17.4 fold increase over untreated cells after 10 days in osteogenic culture (Fig. 4c). Moreover, calcium deposition was enhanced in the cells treated with a single dose of nanoantagomiR-133a, as demonstrated by both quantitative and histological analysis (Fig. 4d,e). This effect corresponded to a 3.36 and a 2.55 fold change over untreated cells respectively at day 10 and 14 (Fig. 4d). Changes in ALP activity induced by manipulation of miRNA levels have been assessed with inhibition of miR-133a resulting in a two-fold increase in ALP activity over untreated cells^33^. From the perspective of this project, the detection of 17-fold increase in ALP activity in the nanoantagomiR-133a group suggested an encouraging enhanced effect that may be associated to the delivery method utilised in our study. Additionally, non-viral delivery RNAi strategies reported a 2-fold increase in calcium deposition by human adipose-derived stem cells (hASCs) after 14 days of osteogenic culture when combining lipoplexes with Chordin siRNA^34^ or miR-148b mimic (40 nM)^35^. Comparatively, the single application of the low dose of 20 nM of nanoantagomiR-133a treatment enhanced calcium deposition above that of lipoplex-based delivery after just 10 days, and remained superior at the endpoint of 14 days, highlighting its significant potential for bone repair.

One surprising result from this study was that negative control miRIDIAN miRNAs, referred to here as scrambled (scr), produced unspecific variations in ALP activity and calcium deposition. A similar effect to that observed in this study was also obtained in terms of calcium deposition when the scr molecules were delivered to the hMSC osteogenic culture using Lipofectamine (data not shown). Of note, scr miRIDIAN controls were titrated to eliminate their effect on ALP activity^33^, and other studies have shown that ALP activity was affected by the treatment with scr miRNAs^30^,^31^. These commercially available controls encompass a cel-miR-67 based sequence and were anticipated to lack targets in mammalian species. However, further BLAST analysis reports 20–30% homology of cel-miR-67 in approximately 100 hits of protein-coding sequences within the human genome, thus possible unspecific interactions may explain this effect. Nevertheless, the effect of the scr sequence was surpassed by the nanoantagomiR-133a treatment, and the specificity of this effect can be related back to the adequate modification of mRNA levels of the direct targets assessed.

In summary, this work demonstrated that nanoantagomiR-133a treatment was capable of enhancing levels of Runx2 and OCN as well as ALP activity, all crucial indicators for osteogenic differentiation. Additionally, and most beneficially from a therapeutic perspective, this study has demonstrated the potent ability of using in-house developed nHA particles to deliver antagomiR-133a to hMSCs resulting in rapidly enhanced calcium production as early as day 10 of the study.

### Analysis of miRNA, mRNA and osteogenic markers along with calcium deposition level in hMSC 3D osteogenic culture

**Figure 5:**
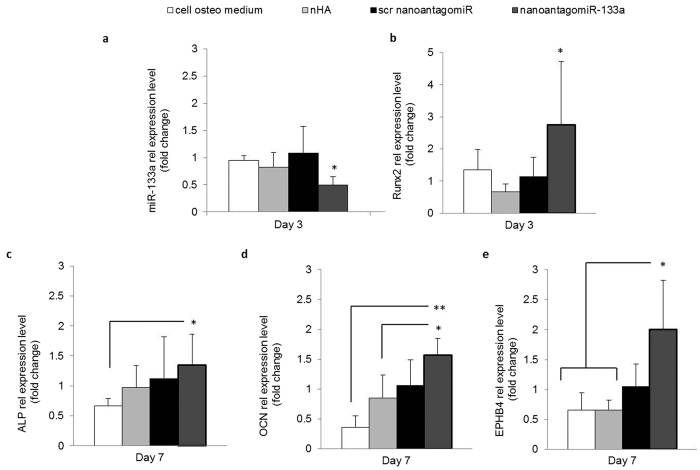
Enhanced osteogenic markers in hMSC 3D culture. (**a**) miR-133a intracellular levels were significantly decreased for hMSCs cultured on the nanoantagomiR-133a activated scaffolds in comparison to the scr activated scaffolds over a timecourse of 14 days, demonstrating a high silencing functionality of the non-viral based 3D delivery system. (**b**) Runx2 mRNA expression was upregulated in the nanoantagomiR-133a activated scaffold group after 3 days. Mean + standard deviation, n = 5, *p < 0.05. (**c**) ALP, (**d**) OCN and (**e**) EPHB4 mRNA expression was upregulated in the nanoantagomiR-133a activated scaffold group after 7 days. Mean + standard deviation, n = 4, *p < 0.05, **p < 0.001.

Having demonstrated the success of the nHA particles as non-viral vectors for microRNA delivery to hMSCs in 2D, subsequently, hMSCs were cultured in the 3D environment of nanoantagomiR-133a activated coll-nHA scaffolds. These 3D platforms significantly decreased the amount of miR-133a available intracellularly in hMSCs to 0.49 ± 0.14 fold after 3 days (Fig. 5a), an effect which was able to trigger a 2.74 ± 1.97 fold change increase in Runx2 mRNA at the same timepoint (Fig. 5b). Importantly, the level of this effect on Runx2 expression can be noted to surpass that achieved by polyethylenimine (PEI)-mediated miR-20a delivery in polyethylenglycol (PEG) hydrogels^7^, at approximately a 2 fold increase, even when the delivery efficiency of PEI has been shown to surpass that of nHA^18^. Furthermore, at a later time point of 7 days, downstream osteogenesis markers including alkaline phosphatase (ALP), OCN and EphrinB4 (EPHB4) were upregulated 1.3, 1.5 and 2 fold respectively (Fig. 5c–e), which was a significant increase in comparison with the levels detected for the cell osteo medium only group. This effect was in line with that reported for CaP-based BMP2 plasmid delivery from titanium mesh scaffolds^35^. Collectively, this data confirmed the ability of coll-nHA scaffolds to mediate a substantially competent manipulation of post-transcriptional gene regulation in hMSC in 3D culture.

**Figure 6:**
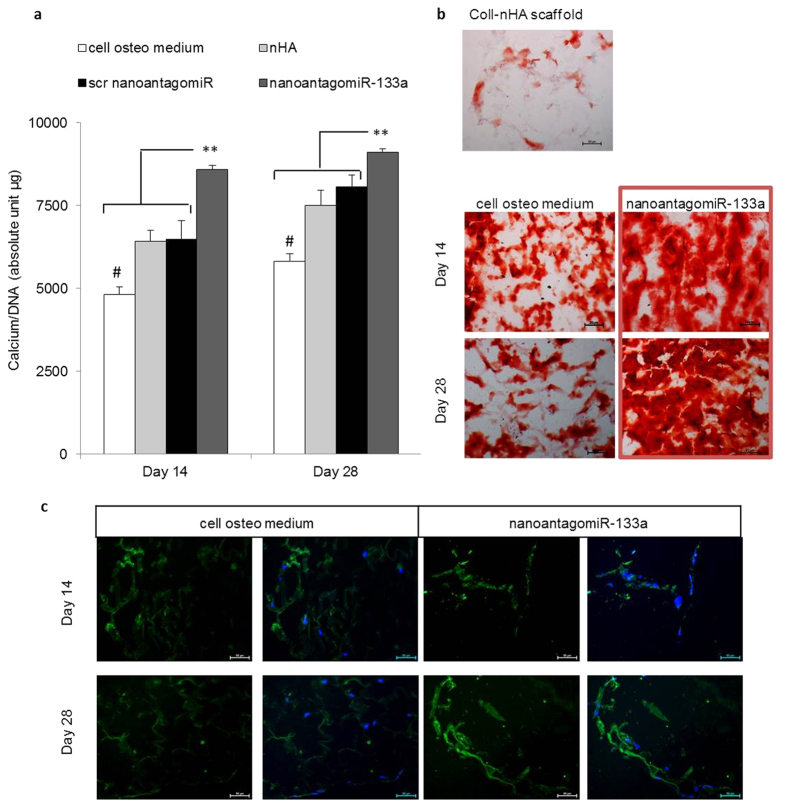
Enhanced mineral matrix deposition in hMSC 3D culture. (**a**) Calcium normalised to dsDNA content confirmed a significant increase in calcium deposition by day 14 in nanoantagomiR-133a activated coll-nHA scaffolds and maintained increased calcium levels compared to the control groups after 28 days. Non cell-seeded scaffolds were used as a control for the determination of calcium presence in the extracellular matrix. Mean + standard deviation, n = 3, **p < 0.001, ^#^p < 0.001 compared to all other groups. (**b**) Alizarin red staining showed increased calcium deposits in the nanoantagomiR-133a loaded coll-nHA scaffold group at 14 and 28 days compared to all other groups. Scale bar = 50 μm. (**c**) OCN immunofluorescence staining (green) after 14 and 28 days in 3D osteogenic culture showed increased protein expression in the nanoantagomiR-133a loaded scaffolds in comparison with the control treatment groups. Nuclei depicted in blue, for DAPI staining, Scale bar = 50 μm.

Similar to the assessment of osteogenesis in monolayer, calcium deposition in the ECM was evaluated at the particularly early timepoint of 14 days for 3D culture, and at 28 days as a final endpoint. Corresponding with the effects of nanoantagomiR-133a activated coll-nHA scaffolds at the gene level, calcium deposition was significantly increased in comparison to all other control groups at both timepoints tested (Fig. 6a), with levels 80% higher than the 3D culture of hMSC in coll-nHA only scaffolds. Moreover, this hallmark effect was also determined using a parallel cell source: rat bone marrow derived MSCs (Supplementary Fig. 1). Histological analysis using alizarin red staining depicted calcium deposition across all groups, which qualitatively increased from the earlier to the later timepoint, and importantly, more prominent, denser staining was found in the nanoantagomiR-133a treated group at both timepoints (Fig. 6b). To further verify the osteogenic differentiation process, the presence of osteocalcin (OCN) at the protein level was assessed by immunofluorescence staining accompanied with DAPI labelling. At both 14 and 28 days after hMSC seeding on the nanoantagomiR-133a activated scaffolds, OCN protein expression was qualitatively increased in comparison to the remaining groups (Fig. 6c). This data correlated with the enhanced levels of OCN mRNA determined for nanoantagomiR-133a activated scaffolds in the gene analysis (Fig. 5d) and collectively pointed to a robust enhancement of hMSC osteogenesis in 3D scaffold culture.

The observation of less pronounced effects in three-dimensionally (3D) cultured MSCs, in comparison to results in monolayer (2D) has been noted in previous reports in the literature^20^,^36^. These studies showed the effect on ALP and OCN expression, as well as on ALP activity, was reduced to approximately half that detected in 2D^37^, while the effect on calcium deposition was reduced to 20%^37^ and 70%^20^ less than in 2D. Remarkably, the reduced calcium deposition from 2D to 3D did not prevent this treatment from enhancing bone repair when tested *in vivo*^20^. A possible explanation for this effect has previously been proposed owing to the 3D scaffold transfection relying on the migration of cells throughout the matrix; thus the larger surface area, in comparison to 2D, would initially reduce the relative exposure of cells to the transfection complexes^38^.

In summary, this data pointed to the successful application of nanoantagomiR-133a for miRNA-mediated osteogenesis of hMSCs using the coll-nHA scaffolds as localised delivery platforms.

Interestingly, out of the panel of over 30 miRNAs described in the literature to be involved in osteogenesis, only 8 have previously been tested as therapeutics to enhance stem cell osteogenesis. In the majority of these the cells internalised the miRNAs in advance of seeding^9^,^10^,^13^,^14^,^38^,^39^, ie. the scaffolds are used to deliver transfected/transduced cells, as opposed to our study, where the scaffolds are used to deliver the miRNAs with a view to transfection of autologous host cells – we contend that this is a major benefit and novelty with our study. Hence, upon implantation in a preclinical rat defect model the rat’s host cells surrounding the defect would infiltrate into the miRNA-activated scaffolds *in vivo*, as similarly approached using pDNA delivery in other studies^18^. Of note, miR-148b has been studied in three different reports involving the use of several delivery methods including both viral and lipid-based vectors^9^,^36^,^38^. While stem cell treatment with miR-148b mimics elicited promising therapeutic effects and was linked to triggering enhanced ALP activity, the silencing of a direct target involved in the osteogenic process has not been experimentally validated for this miRNA. Similarly, the direct osteogenic target validated for miR-26a, SMAD1, was not studied when miR-26a was assessed for bone repair. Although markers of both osteogenesis and angiogenesis were found to be increased in this study, the molecular course of activation of such processes was not examined^14^. Additionally, it is important to note that miRNAs tested as osteo-therapeutics so far, with the exception of miR-31, were not validated to target key activators of the osteogenesis pathway, but rather intermediate signalling molecules which are part of side signalling pathways in many cases^12^. Contrary to this, the innovative approach evaluated in this study focussed on miRNA-mediated manipulation of the levels of Runx2, the transcription factor regarded as the primary driver of the osteogenic pathway. Moreover, by incorporating nanoantagomiR-133a complexes in the 3D scaffolds in a cell-free manner, the coll-nHA scaffolds were utilised as reservoirs for the localised delivery of the miRNA complexes to the cells, with an associated potential to exist as ‘off-the-shelf’ platforms for bone repair. Future assessment of this system in a pre-clinical animal model of bone defect will inform about the potential of this system to improve the regeneration of bony tissue.

Taken together, this study has produced an innovative alternative to existing bone graft treatments and represents a promising new concept in tissue engineering through the inhibition of miR-133a in hMSCs for the first time using hydroxyapatite based delivery on porous collagen-based scaffolds. The results of this study showed enhanced stem cell mediated osteogenesis using scaffolds that were miRNA-activated in advance of cell seeding, pointing to the exciting potential of further enhancing the therapeutic application of a biomaterial previously optimised for bone repair applications. Incorporation of the nanoantagomiR-133a treatment in these 3D platforms showcased a successful example of enhanced hMSC osteogenesis through the novel application of a miRNA-based strategy focussed directly on a key activator of osteogenesis, Runx2. The significant therapeutic efficiency achieved with the use of the beneficial non-viral nHA particles additionally indicated that this vector should not be overlooked in the derivation of new therapeutics in the field. Importantly from an overall perspective, since the nanomiR activated scaffolds continue to exert successful manipulation of MSC gene expression, combining this platform technology with miRNAs orchestrating distinct pathways in other tissues opens a wide avenue of application to other therapeutic areas beyond bone repair. Moreover, these scaffolds can be tailored for delivery of numerous miRNA cargos demonstrating the potential applicability of this platform for a myriad of applications, including as advanced 3D pathophysiology *in vitro* systems for disease modelling, as systems for drug discovery or analysis of drug transport and function- thus presenting a new paradigm for both tissue engineering and across the multidisciplinary fields of biomedicine and drug development.

## Methods

### nanohydroxyapatite (nHA) - miRNA (nanomiR) and miRNA-activated scaffold systems

nHA particles were synthesised following an *in situ* precipitation protocol established previously^20^. Briefly, a phosphate solution (12 mM), containing 0.017% (V/V) Darvan 821A dispersant reagent (RT Vandervilt), was added to an equal volume of calcium chloride solution (20 mM) and filtered through a 0.2 μm filter^20^. nHA particles (150 μl) were added to a scrambled (scr) or hsa-miR-133a miRIDIAN antagomiR solution (Dharmacon) prepared at a final 20 nM concentration per well, following the method developed in-house^19^. Collagen-nHA (coll-nHA) scaffolds were manufactured using a freeze-drying technique as previously reported^23^,^40^,^41^. Briefly, coll-nHA suspensions were prepared at a 1:1 weight ratio by homogenising in-house synthesised nHA particles and collagen type I (Integra Life Sciences) within an acetic acid (0.05 M) solution. Coll-nHA suspensions were lyophilised at a final freezing temperature of −40 °C using a VirTis Genesis 25 EL freeze-dryer (Biopharma). Scaffolds were then subjected to dehydrothermal sterilisation, cut into cylindrical discs (8 mm × 4 mm), and chemically cross-linked with a solution of 14 mM 1-(3-Dimethylaminopropyl)-3-ethylcarbodiimide hydrochloride (EDAC, Sigma-Aldrich) and 5.5 mM N-hydroxysuccinimide (NHS, Sigma-Aldrich). Finally, coll-nHA scaffolds were soak-loaded on both sides with *in situ* prepared nanomiRs (150 μL total at a 20 nM concentration).

### Human mesenchymal stem cell (hMSC) culture

hMSCs from iliac crest bone marrow aspirates of healthy human volunteers were kindly provided by REMEDI Galway; all experimental procedures were approved by the Clinical Research Ethical Committee at University College Hospital, Galway, and written informed consent was obtained from all subjects. All the bone marrow aspirates were treated in accordance with all the relevant guidelines and regulations, and stringent analysis of cell phenotype was carried out in accordance with all the relevant guidelines, following which hMSCs were obtained^42^. Cells were cultured using low-glucose DMEM (Sigma), supplemented with foetal bovine serum (FBS; 10%, Sigma) plus penicillin/streptomycin (1%, Sigma) and regularly cleared for mycoplasma contamination tests. Cells (passage 4–6) were used at seeding densities of 3 × 10^4^ cells per well (6 well plates) for monolayer, and 3 × 10^5^ cells per scaffold for 3D experiments. Complete osteogenic media consisted of standard growth medium supplemented with 50 μg/mL ascorbic acid-2-phosphate, 10 nM β-glycerophosphate and 100 nM dexamethasone.

### Quantitative Real Time Polymerase Chain Reaction (qRT-PCR)

qRT-PCR was used to determine levels of miR-133a, Runx2, and OCN after transient transfection using the nanomiR method. Briefly, total RNA extraction was performed using QIAzol plus a miRNeasy kit (Qiagen) under manufacturer’s instructions. The Quantitect Reverse Transcription kit combined with the validated Quantitect primer assays (Qiagen) plus SYBR Green master mix (Roche) was applied for the measurement of mRNA levels, and the Taqman miR-PCR assay kit (BioSciences) for miR-133a levels. Relative expression normalised to 18S ribosomal RNA was calculated using the 2(-ΔΔCt) method with scr nanomiR treatments set as the reference group.

### Mineral deposits quantification

A Calcium Liquicolor kit (Stanbio Laboratories) was used under manufacturer’s instructions for calcium quantification. Calcium content quantified for blank coll-nHA scaffolds (non cell-seeded) was subtracted from the content determined for all cell-seeded groups. Absorbance of the colour product was read using a Varioskan Flash plate reader (ThermoScientific). Complementarily, the dsDNA Quant-iT PicoGreen kit (BioSciences) was used under manufacturer’s instructions using the Varioskan system as before.

### Histological assessment of calcium deposition

Monolayer samples were fixed using 10% formalin and stained with 2% Alizarin red staining. Images were captured using the Leica - LAS V3.6 imaging system (Leica). Scaffolds were fixed using 10% formalin and dehydrated using an automatic tissue processor (ASP300, Leica) prior to paraffin wax embedding. Serial sections were prepared for histological analysis and rehydrated prior to staining with 2% Alizarin red. Digital imaging was carried out using a microscope system (Eclipse 90i plus DS Ri1, Nikon) coupled to NIS Elements software.

### Osteocalcin immunofluorescence staining

Scaffold sections were permeabilised in 1% Triton X100 and blocked using 5% horse serum. Samples were incubated with rabbit polyclonal IgG anti-osteocalcin antibody (1:50), FITC- goat anti-rabbit IgG antibody (1:200) (both from Santa Cruz Inc.) and mounted with Fluoroshield-DAPI (Invitrogen). Digital imaging was carried out using the Nikon microscope system as before and Image J software was utilised to generate merge images.

### Statistical analysis

Experiments were performed in triplicate, unless otherwise specified within figure captions, and are representative of a minimum of three independent repetitions using two cell donors. Sample size was chosen based on established procedures in the lab for this delivery system^19^ and application^17^,^18^. qRT-PCR Ct values were subjected to a Grubb’s test for outliers exclusion with 95% confidence interval. SigmaPlot 11.0 software was used to perform analysis of variance (ANOVA) plus a Tukey *post-hoc* test following software in-built data normality analysis. Two-way of ANOVA was carried out for data studied at several timepoints and one-way ANOVA for data assessed at a singular timepoint. p < 0.05 and p < 0.001 were considered significant.

## Additional Information

**How to cite this article**: Castaño, I. M. *et al.* Next generation bone tissue engineering: non-viral miR-133a inhibition using collagen-nanohydroxyapatite scaffolds rapidly enhances osteogenesis. *Sci. Rep.* **6**, 27941; doi: 10.1038/srep27941 (2016).

## Supplementary Material

Supplementary Information

## Figures and Tables

**Figure 1:**
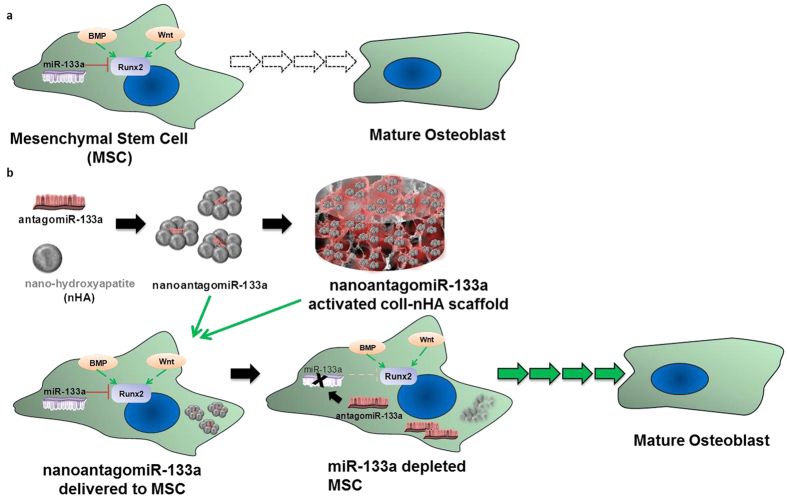
AntagomiR-133a role in mesenchymal stem cell (MSC) osteodifferentiation. (**a**) Extracellular ligands such as Bone Morphogenetic Proteins (BMPs) and wingless-related integration site family (Wnt) proteins initiate complex signaling pathways (green arrows) that activate the Runt-related transcription factor 2 (Runx2) to initiate differentiation towards a mature osteoblast state, while miR-133a specifically targets and inhibits (red brake symbol) Runx2. (**b**) AntagomiR-133a forms complexes with nHA particles which are either delivered to MSCs directly or on porous collagen-nHA scaffolds. They bind to and inhibit miR-133a (black arrow and X symbol), diminishing the silencing of Runx2 (faded red brake symbol), which results in higher availability of functional levels of Runx2. Runx2 drives MSCs along the osteogenic lineage in progressive maturity stages.
