# Supplementary material for: Next generation bone tissue engineering: non-viral miR-133a inhibition using collagen-nanohydroxyapatite scaffolds rapidly enhances osteogenesis
Source: Sci Rep. 2016 Jun 14;6:27941. doi: 10.1038/srep27941 (PMC4906381; doi:10.1038/srep27941)
Supplement: Supplementary Information [file srep27941-s1.doc]

**SUPPLEMENTARY INFORMATION**

**Next generation bone tissue engineering: non-viral miR-133a inhibition using collagen-nanohydroxyapatite scaffolds rapidly enhances osteogenesis**

*Irene Mencía Castaño, Caroline M. Curtin, Garry P. Duffy & Fergal J. O’Brien*


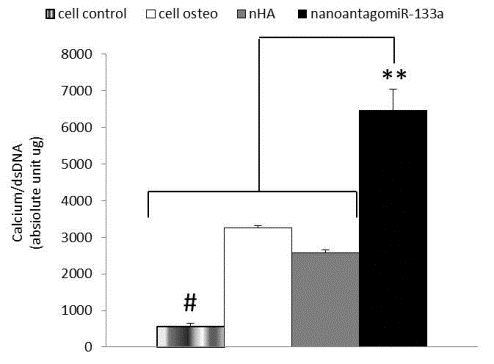


**Supplementary Figure 1.** Calcium normalised to dsDNA content confirmed an increase in calcium deposition by rat mesenchymal stem cells grown in nanoantagomiR-133a loaded coll-nHA scaffolds compared to the control groups after 14 days. Mean + standard deviation, n=3, ** = p<0.001, #=p<0.001 compared to all other groups.
